# Competitive Analysis of the Online Leasing Problem for Scarce Resources

**DOI:** 10.3390/ijerph20010886

**Published:** 2023-01-03

**Authors:** Jiamin Lu, Nishan Chen, Xin Feng

**Affiliations:** College of Economics and Management, Nanjing Forestry University, Nanjing 210037, China

**Keywords:** online leasing problem, competitive analysis, scarce resource, low-carbon economy

## Abstract

The leasing activities of enterprises are of positive significance for promoting a green and low-carbon economy. For scarce resources that can easily go into tight supply states due to changes in the external market environment, the sudden change of their price is a common phenomenon in the leasing market. This paper provides an online leasing model based on the characteristics of scarce resources in which the resource might have a sudden price surge, and the length of its usage time cannot be known in advance. An online leasing strategy *ON* was then developed to achieve the minimum possible acquisition cost for the scarce resource. This strategy was proven to be the optimal online strategy through competitive analysis. Computational experiments were conducted to evaluate the performance of the proposed online strategy. Furthermore, sensitivity analysis of the problem parameters shows that increasing resource reserves and reducing the price-to-rent ratio are effective ways to improve the performance of enterprise leasing operation management for scarce resources.

## 1. Introduction

As a part of sharing economy, the leasing industry can both revitalize the stock assets for enterprises and reduce resource consumption to promote low-carbon transformation [1,2,3]. If an enterprise needs a specific resource for daily operation, instead of paying a large amount of cost to buy the resource directly, it can obtain the usage right of the resource from a leasing company by paying a small amount of periodic rental fee. In this way, the leasing industry can help an enterprise assure steady cash flow, spread risk, and reduce the cost of capital [4,5]. In addition, compared with purchasing behavior, leasing can effectively avoid wasting phenomena with a lot of resources left unused [6].

In recent years, drastic changes, such as trade frictions, military conflict between countries, or stringent environmental and resource protection policies, have significantly impacted the supply of some specific resources, especially scarce ones [7,8,9,10]. For example, the military conflict between Russia and Ukraine in 2022 led to a serious shortage of natural gas resources, and the outbreak of COVID-19 led to a shortage of medical resources, such as masks and ventilators, in the first half of 2020. Therefore, the supply of scarce resources can be easily threatened due to some accidental incidents even under normal circumstances and thus might lead to a soaring price due to a tight supply. The price surge of scarce resources also brings challenges to the leasing industry [11]. Against this background, how to make decisions on acquiring scarce resources with the minimum cost through the leasing industry has become one of the critical problems for enterprises with urgent needs to ease cash flow pressures or achieve low carbon transformations.

Generally speaking, to decide if leasing is a profitable financing option, the decision maker should know both the price and the usage time of the resources [12,13]. However, in practice, the usage time is often impossible to be known in advance. In addition, the unsteady supply of scarce resources intensifies the uncertainty of the decision-making environment. Hence, an online version of the leasing decision problem arises in such a scenario, which is known as the online leasing problem (a.k.a. ski rental problem). Because of the uncertainties, we adopt a competitive analysis method to evaluate the online leasing decision with a competitive ratio. Such a technique compares the results obtained with an online strategy to the result that could have been obtained if one had known the exact length of usage time in advance, with the latter scenario represented by an optimal offline strategy. The competitive analysis method has been widely applied in the fields of finance [14,15], operations research [16,17,18,19], and computer science [20,21].

This paper studies an online leasing problem for scarce resources, which is a variant of the classical online leasing problem, by considering possible soaring prices due to supply changes. The classical online leasing problem was first studied by [22], where the decision maker has two options to acquire the resource, i.e., to lease or purchase the resource. If the resource is acquired by the lease option, its using cost increases with the lease period; Once it is purchased, a one-time cost is incurred, and thereafter, usage is free of charge. An optimal online strategy with a competitive ratio of 2 is proposed in [22], i.e., the online strategy never pays more than twice the optimum.

After that, an abundant amount of research extends the classical online leasing problem to illustrate real situations, such as online leasing problems considering interest rates [23,24,25], discounts [26,27,28], down payments [29], and separable properties [30]. In addition, variants of lease options in the online leasing problem are also investigated in [12,31,32]. Khanafer et al. [33] further factored in the availability of statistical data on the usage time of the resource to perform smarter decision making. For other classic variations of the online leasing problem, we refer readers to the replacement problem [34], the capital investment problem [35], and the Bahncard problem [36].

In all of the above literature, the resource price is assumed to be unchanged. The online leasing problem considering time-varying prices is investigated in [13,37,38,39,40,41,42,43]. Irani and Ramanathan [37] considered a situation in which the resource price lies in a predetermined interval [1, *M*] while the rental fee remained unchanged. Bienkowski [38] considered another online leasing problem where the prices on two consecutive days differed by at most 1. After that, the online leasing problem with price fluctuation bounded by CPI is studied in [13,39,40], in which the ratio between prices on two consecutive days was strictly less than a given upper bound. In addition, online leasing problems with depreciable resources are investigated in [41,42,43].

In essence, the online leasing problem for scarce resources studied in this paper also belongs to the online leasing problem with time-varying prices. Different from the above literature that focuses on daily changes in resource prices, the required resources in this paper may undergo drastic supply changes, and, therefore, be known as scarce resources, whose characteristics are mainly reflected by a sudden change of prices.

The remainder of the paper is organized as follows: In Section 2, we define the online leasing problem with scarce resources. In Section 3, an online leasing strategy *ON* and its competitive analysis are proposed. Section 4 derives the lower bound on the competitive ratio of the studied problem. Numerical experiments are presented in Section 5. Section 6 presents the conclusions and the direction of future work.

## 2. Problem Definition and Notation

In this section, we present the precise definition of the online leasing problem for scarce resources.

Formally, for an enterprise (lessee) seeking a specific scarce resource for daily operation, it can acquire the resource usage rights by lease or purchase option. That is to say, the enterprise can lease the resource for a rental fee per unit of time, or it can buy the resource by paying a one-time cost. Because of the scarcity, the resource has two supply states, i.e., regular supply and tight supply. In the regular supply state, the lessee can lease the resource at a unit rental fee, or it buy the resource at the price of P≥1. When the resource turns into a tight supply state, its price rises because of scarcity. By assuming that the price-to-rent ratio remains unchanged, the rental fee and the price are increased to be *α* and *αP*, respectively, where α≥1 the price increase range reflects the inadequacy of the scarce resource. In addition, the following basic assumptions on the uncertainties are used in this paper:

(1) The length of time for use of the scarce resource is unknown;
(2) The supply of the scarce resource cannot be known in advance, i.e., the price and the rental fee of the resource at time t cannot be known until that time.

Let *C_ON_* be the cost for using the resource produced by an online strategy *ON.* To evaluate the online strategy *ON*, *C_ON_* is compared with the cost obtained by a so-called optimal offline strategy, which knows all the information on the usage time and the supply states in advance. The corresponding cost for using the resource produced by the optimal offline strategy is defined as *C_opt_*.

The enterprise always wants to find the best acquisition strategy for a scarce resource with minimal cost. However, in the online version of the problem, neither the usage time nor the supply state of the resource can be known in advance. A well-established technique, i.e., online optimization, is explored to deal with this type of uncertainty. The performance of an online strategy *ON* is generally evaluated by the competitive ratio [44]. Translated into the terminology of our problem, the strategy *ON* is *ρ*-competitive if $C_{ON}\leq \rho C_{opt}+\varepsilon$ holds for any possible scenarios, where *ρ* is a given constant, and *ε* is an arbitrary real number. The ratio *ρ* is also called the competitive ratio of the online strategy *ON*. Thus, the aim of this paper is to acquire the scarce resource with a minimal competitive ratio *ρ* for any possible scenarios. The parameters used in this paper are summarized in Table 1.

## 3. Online Leasing Strategy

In this section, we examine the online leasing problem with scarce resources and propose an online strategy *ON*.

We use *r_t_* to denote the rental fee of the scarce resource at time *t*, and then the price of the scarce resource at that time is *Pr_t_.* Note that *r_t_ =* 1/*P* if the scarce resource is in the regular supply state at time *t.* Otherwise, *r_t_* = *α/P* if the scarce resource is in the tight supply state. Let *R_t_* denote the cumulative amount spent on rental fees if the purchase option does not occur before time *t*, where *R*_1_ = 0. In addition, because of the possible price soaring due to scarcity, a threshold *λ* < 1 is introduced in the proposed online strategy *ON* in this paper. Using parameters *R_t_* and *λ*, the online strategy *ON* is proposed as follows:**Online Strategy *ON***Keep leasing the resource and switch to the purchasing option at time *t*, where *t* satisfies the condition $R_{t}+r_{t}\geq \lambda P$.

In the online strategy *ON*, the exact value of *λ* will be derived from the following discussion in the competitive analysis.

Now that the online leasing strategy *ON* has been proposed, we need to derive its competitive ratio through a competitive analysis. The competitive analysis is carried out based on the length of the resource usage time and the value of *λ*.

Denote the length of the resource usage time as *L* if *L* is comparatively short so that the total rental fee paid for the resource until time *L* is less than λP, i.e., $R_{L}+r_{L}\leq \lambda P$. According to the online strategy *ON*, the lessee keeps leasing the resource during the entire usage time. In this case,

(1) $$C_{ON}=R_{L}+r_{L}$$

Because $P>\lambda P\geq R_{t}+r_{t}$, the optimal offline strategy also keeps leasing the resource during the entire usage time with the optimal cost as

(2) $$C_{opt}=R_{L}+r_{L}$$

According to (1) and (2), *C_ON_* = *C_opt_*, which implies the online strategy *ON* is the best online strategy in the case when $R_{L}+r_{L}<\lambda P$.

Otherwise, *L* is long enough so that $R_{L}+r_{L}>\lambda P$, which implies that there exists a time τ<L that satisfies $\tau =\max\left\{t|R_{t}+r_{t}\leq \lambda P\right\}$. In this case, we discuss the competitive ratio of the online strategy *ON* by the value of the threshold parameter *λ*.

**Lemma 1.**  *When*$\lambda P\leq R_{\tau }+r_{\tau }$, $\frac{C_{ON}}{C_{opt}}\leq 1+\frac{P-1}{\lambda }\cdot \frac{\alpha }{P}$.

**Proof of Lemma 1.**  According to the online strategy ON, when $\lambda P\leq R_{\tau }+r_{\tau }$, the enterprise purchases the resource at time *τ*. The corresponding cost is

(3) $$C_{ON}=R_{\tau }+P\cdot r_{\tau }$$

While for the optimal offline strategy, if it buys the resource at time t<τ,

(4) $$C_{opt}\geq P\cdot r_{t}\geq P\geq R_{\tau }+r_{\tau }$$

Otherwise, if the purchasing option is activated no earlier than *τ*, then

(5) $$C_{opt}\geq R_{\tau }+r_{\tau }$$

According to (4) and (5), when the threshold *λ* is set low so that $\lambda P\leq R_{\tau }+r_{\tau }$, the optimal cost for using the resource is $C_{opt}\geq R_{\tau }+r_{\tau }$.

In this case, the ratio between the costs paid by the online strategy ON and the optimal offline strategy is

(6) $$\frac{C_{ON}}{C_{opt}}\leq \frac{R_{\tau }+P\cdot r_{\tau }}{R_{\tau }+r_{\tau }}=1+\frac{\left(P-1\right)r_{\tau }}{R_{\tau }+r_{\tau }}\leq 1+\frac{\left(P-1\right)r_{\tau }}{\lambda P}\leq 1+\frac{\left(P-1\right)\alpha }{\lambda P}$$

The lemma is proved. □

**Lemma 2.**  *When*$\lambda P>R_{\tau }+r_{\tau }$, $\frac{C_{ON}}{C_{opt}}\leq \lambda +\alpha$.

**Proof of Lemma 2.**  According to the definition of *τ*, $R_{\tau }+r_{\tau }<\lambda P<P<R_{\tau +1}+r_{\tau +1}$. Hence, the enterprise buys the resource at time *τ* + 1. The corresponding cost is

(7) $$C_{ON}=R_{\tau +1}+P\cdot r_{\tau +1}=R_{\tau }+r_{\tau }+P\cdot r_{\tau +1}$$

While for the optimal offline strategy, if it buys the resource at time t<τ+1,

(8) $$C_{opt}\geq P\cdot r_{t}\geq P$$

Otherwise, the purchasing option is activated no earlier than τ+1,

(9) $$C_{opt}\geq R_{\tau +1}+r_{\tau +1}\geq P$$

Hence, when the threshold *λ* is set high so that $\lambda P>R_{\tau }+r_{\tau }$, the optimal cost for using the resource is $C_{opt}\geq P$.

In this case, the ratio between the costs paid by the strategy *ON* and the corresponding optimal offline strategy is

(10) $$\frac{C_{ON}}{C_{opt}}\leq \frac{\lambda P+P\cdot r_{\tau +1}}{P}=\lambda +r_{\tau +1}\leq \lambda +\alpha$$

The lemma is proved. □

According to Lemmas 1 and 2, when the length of the resource usage time *L* is long enough so that $R_{L}+r_{L}>\lambda P$, the ratio between the costs paid by the strategy *ON* and the optimal offline strategy depends on the value of *λ*. In other words, through the selection of the preferred value of the threshold *λ*, the online strategy *ON* can achieve a competitive ratio as

(11) $$\rho =\max\left\{1+\frac{\left(P-1\right)\alpha }{\lambda P},\lambda +\alpha \right\}$$

Let $1+\frac{\left(P-1\right)\alpha }{\lambda P}=\lambda +\alpha$; the preferred value of *λ* can be determined as $\lambda =\lambda^{*}=\frac{\sqrt{{\left(\alpha -1\right)}^{2}P^{2}+4\alpha P\left(P-1\right)}-\left(\alpha -1\right)P}{2P}$, and the competitive ratio of the strategy *ON* is $\rho =\sqrt{\frac{P{\left(\alpha +1\right)}^{2}-4\alpha }{4P}}+\frac{\alpha +1}{2}$. Therefore, the following theorem is established.

**Theorem 1.**  *When*$\lambda =\frac{\sqrt{{\left(\alpha -1\right)}^{2}P^{2}+4\alpha P\left(P-1\right)}-\left(\alpha -1\right)P}{2P}$*, the competitive ratio of the strategy ON is*$\rho =\sqrt{\frac{P{\left(\alpha +1\right)}^{2}-4\alpha }{4P}}+\frac{\alpha +1}{2}$.

## 4. Lower Bound

To measure the gap between the online strategy *ON* and the optimal online strategy, it is necessary to derive a lower bound on the competitive ratio of online leasing problems with scarce resources.

For the online leasing problem with scarce resources, we define an online strategy as *A*(*t*) if it switches the lease option to the purchase option at time *t*. Then Theorem 2 is established to derive the lower bound.

**Theorem 2.**  *The lower bound on the competitive ratio of online leasing problems with scarce resources is*$\sqrt{\frac{P{\left(\alpha +1\right)}^{2}-4\alpha }{4P}}+\frac{\alpha +1}{2}$.

**Proof of Theorem 2.**  To prove the theorem, it suffices to construct an instance to make the online strategy *A*(*t*) perform poorly, which has no competitive ratio less than $\sqrt{\frac{P{\left(\alpha +1\right)}^{2}-4\alpha }{4P}}+\frac{\alpha +1}{2}$ for any value of *t*. The instance is constructed by an offline adversary who knows everything about the future.

At the beginning, the offline adversary set the resource in the regular supply state, i.e., the price of the resource is *P* at time t=1. After that, the resource remains in the tight supply state, i.e., the price of the resource is *αP* at time t>1. For the online strategy A(t), we investigate two cases of t to derive the lower bound of the online leasing problem with scarce resources.

Case 1: t≤τ. In this case, the offline adversary terminates the usage time of the resource at time *t*, i.e., L=t. Then,

(12) $$C_{A\left(t\right)}=\left\{ \begin{array}{ll} 1+\left(t-2\right)\alpha +\alpha P & t\geq 2\\ P & t=1 \end{array}\right.$$

While for the optimal offline strategy, it keeps leasing the resource until time *t*. The corresponding cost is

(13) $$C_{opt}=\left\{ \begin{array}{ll} 1+\left(t-1\right)\alpha & t\geq 2\\ 1 & t=1 \end{array}\right.$$

According to (12) and (13), when t≤τ,

(14) $$\frac{C_{A\left(t\right)}}{C_{opt}}=\left\{ \begin{array}{ll} \frac{1+\left(t-2\right)\alpha +\alpha P}{1+\left(t-1\right)\alpha } & t\geq 2\\ P & t=1 \end{array}\right.$$

Because α≥1, when t≥2,

(15) $$\frac{1+\left(t-2\right)\alpha +\alpha P}{1+\left(t-1\right)\alpha }=1+\frac{\alpha \left(P-1\right)}{1+\left(t-1\right)\alpha }\leq 1+\frac{\alpha \left(P-1\right)}{1+\alpha }\leq P$$

According to (14) and (15), when t≤τ,

(16) $$\begin{array}{ll} \frac{C_{A\left(t\right)}}{C_{opt}} & \geq \frac{1+\left(t-2\right)\alpha +\alpha P}{1+\left(t-1\right)\alpha }\\ & \geq \frac{1+\left(\tau -2\right)\alpha +\alpha P}{1+\left(\tau -1\right)\alpha }\\ & =1+\frac{\alpha \left(P-1\right)}{1+\left(\tau -1\right)\alpha }\\ & \geq 1+\frac{\alpha \left(P-1\right)}{\lambda^{*}P} \end{array}$$

Case 2: t>τ. In this case, the offline adversary terminates the usage time of the resource at time L=+∞. Then,

(17) $$C_{A\left(t\right)}=1+\left(t-2\right)\alpha +\alpha P$$

While for the optimal offline strategy, it purchases the resource at the beginning time. The corresponding cost is

(18) $$C_{opt}=P$$

According to (17) and (18), when t>τ,

(19) $$\begin{array}{ll} \frac{C_{A\left(t\right)}}{C_{opt}} & =\frac{1+\left(t-2\right)\alpha +\alpha P}{P}\\ & \frac{1+\left(\tau -2\right)\alpha +\alpha P}{P}\\ & =\frac{\lambda^{*}P+\alpha P}{P}\\ & \geq \lambda^{*}+\alpha \end{array}$$

From the above two cases, it can be concluded that

(20) $$\frac{C_{A\left(t\right)}}{C_{opt}}\geq \left\{ \begin{array}{ll} 1+\frac{\alpha \left(P-1\right)}{\lambda^{*}P} & t\leq \tau \\ \lambda^{*}+\alpha & t>\tau \end{array}\right.$$

As mentioned in Section 3, $\rho =\sqrt{\frac{P{\left(\alpha +1\right)}^{2}-4\alpha }{4P}}+\frac{\alpha +1}{2}=1+\frac{\left(P-1\right)\alpha }{\lambda^{*}P}=\lambda^{*}+\alpha$ when $\lambda^{*}=\frac{\sqrt{{\left(\alpha -1\right)}^{2}P^{2}+4\alpha P\left(P-1\right)}-\left(\alpha -1\right)P}{2P}$. Therefore, for any online strategy A(t), $\frac{C_{A\left(t\right)}}{C_{opt}}\geq \rho$. In other words, the lower bound on the competitive ratio of online leasing problems with scarce resources is $\rho =\sqrt{\frac{P{\left(\alpha +1\right)}^{2}-4\alpha }{4P}}+\frac{\alpha +1}{2}$. The theorem is proved. □

According to Theorems 1 and 2, the competitive ratio of the online strategy *ON* equals the lower bound on the competitive ratio of the problem. Therefore, the online strategy *ON* is the optimal strategy for the online leasing problem with scarce resources.

More specifically,

(21) $$\frac{\partial \rho }{\partial P}=\frac{\alpha }{2P^{2}}{\left(\frac{{\left(\alpha +1\right)}^{2}}{4}-\frac{\alpha }{P}\right)}^{-\frac{1}{2}}>0$$

(22) $$\frac{\partial \rho }{\partial \alpha }=\frac{1}{2}\left(\frac{\alpha +1}{2}-\frac{1}{P}\right){\left(\frac{{\left(\alpha +1\right)}^{2}}{4}-\frac{\alpha }{P}\right)}^{-\frac{1}{2}}+\frac{1}{2}>0$$

Based on (21) and (22), it can be concluded that both the price-to-rent ratio (*P*) and the price in the tight supply state (*α*) are positively correlated with the competitive ratio of the optimal online strategy. Hence, a high price-to-rent ratio or a high price in a tight supply state worsens the operational situation for the enterprise using scarce resources for daily operations. In the next section, more detailed performance analyses of the proposed online strategy and managerial implications are presented through numerical experiments.

## 5. Numerical Experiments

In this section, numerical experiments are conducted to provide more detailed guidance for enterprises seeking scarce resources under an unstable supply state.

### 5.1. Sensitivity Analysis

**Figure 1:**
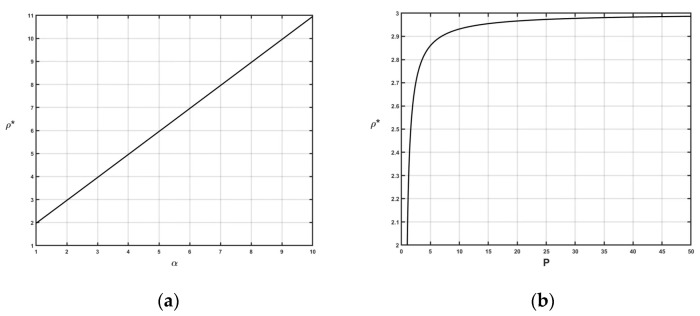
Illustration of the sensitivity analysis results. (**a**) The influence of the price increase range; (**b**) The influence of the price-to-rent ratio.

**Figure 2:**
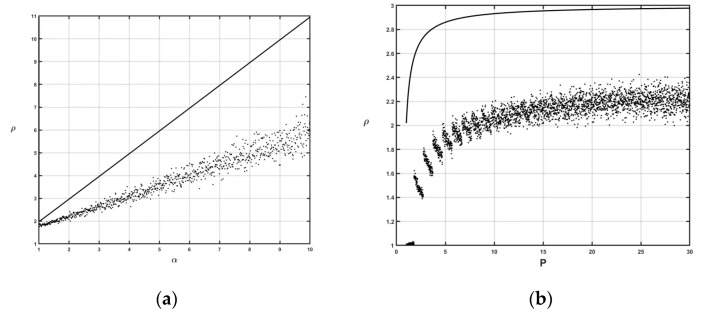
Average- and worst-case performance of the online strategy *ON.* (**a**) α={1,   1.01,   1.02,⋯,   10} and *P* = 20. (**b**) α=2 and P={1,   1.1,   1.2,⋯,   30}.

In this subsection, two sets of numerical experiments are conducted to identify the impact of the parameters, i.e., the price increase range *α* and the price-to-rent ratio *P*, on the online strategy performance. For a better understanding, the trend of the results obtained by online strategy *ON* for different parameters is illustrated in Figure 1 and Figure 2.

Figure 1a shows that the competitive ratio of online strategy *ON* is positively correlated with *α*. Note that the competitive ratio reflects the cost gap between online and offline environments. A higher competitive ratio implies a poor ability to hedge against uncertainties. Therefore, a high price increase range *α* in the tight supply state worsens the operational situation for enterprises acquiring resources from the leasing industry. The price increase range mainly depends on the tightness of the supply state. For a scarce resource, even if it is cheap in the regular supply state, it could undergo an extreme price increase range in the tight supply state, thereby deteriorating the operational situation. Hence, a stable supply is more important than the price for the scarce resource in the leasing market. The leasing company should establish reliable relationships with adequate suppliers to assure a stable supply.

Furthermore, it can be observed from Figure 1b that the competitive ratio is also positively correlated with *P*, which implies that a high price-to-rent ratio aggravates the negative impact of uncertainties on the leasing market. Especially, the competitive ratio grows rapidly with *P* when *P* ≤ 5 and approaches abysmally close to an upper bound when *P* > 5. From the mathematical point of view, this is because the competitive ratio is a hyperbolic function of *P*. When *P* is large enough, the competitive ratio goes to an upper bound limit, i.e., $\underset{P\to \infty }{\lim}\rho =\underset{P\to \infty }{\lim}\left(\sqrt{\frac{P{\left(\alpha +1\right)}^{2}-4\alpha }{4P}}+\frac{\alpha +1}{2}\right)=\alpha +1$. From a practical point of view, according to the *ON* strategy, a resource with a larger price-to-rent ratio is more inclined to be rented for a longer time before it is purchased. Because the lease option cannot obtain the ownership of the resource, and thus is more likely to be affected by the changing supply, a larger value of the price-to-rent ratio thereby leads to a more significant negative impact of uncertainties. Therefore, governments should carry out market adjustments to keep the price-to-rent ratio within a reasonable range.

### 5.2. Average-Case Performance Evaluation

Because the competitive ratio only reflects the strategy’s performance under worst-case scenarios, it is necessary to derive strategy performances under average-case scenarios, which can verify the effectiveness of the proposed online strategy in reality more comprehensively. To evaluate the performance of the online strategy *ON* under average-case scenarios, a group of numerical experiments involving randomly generated instances was conducted. For all the tested instances, the price-to-rent ratio *P* and the price increase range *α* were generated based on [13]. Specifically, the price-to-rent ratio was set as *P =* {1, 1.1, 1.2, …, 30} and *α =* {1, 1.01, 1.02, …, 10}. For each instance when *α* was determined, the length of the usage time, i.e., *L*, took a value from the interval [1, 100] randomly. In addition, the supply of the scarce resource was set to be regular or tight randomly each time. In this way, when the supply and the length of the usage time of the scarce resource were both determined, the optimal cost *C_opt_* could be easily calculated by at most 100 enumerations (i.e., the 100 possible times at which the lessee could switch from the lease to the purchase). In addition, ten replications of the instances were generated for each combination of *P* and *α* to eliminate randomness, which resulted in a total of 300,000 tested instances. The computational results are shown in Figure 2.

As shown in Figure 2, the average-case cost ratio between the online strategy *ON* and the optimal offline strategy is only about half of the competitive ratio. According to Figure 2a, the cost ratio under the average-case scenario varies from 1.7 to 6.6 when *P* = 20 and *α* grows to 10 while the competitive ratio under the worst-case scenario grows rapidly from 1 to 11. Similarly, Figure 2b shows that the average-case cost ratio is also significantly less than the competitive ratio when *α* = 2 and *P* grows to 30. Therefore, although the online strategy *ON* has a competitive ratio of $\sqrt{\frac{P{\left(\alpha +1\right)}^{2}-4\alpha }{4P}}+\frac{\alpha +1}{2}$, it can help enterprises to achieve a much better management performance for scarce resource acquisition in real situations.

## 6. Conclusions

As people attach more importance to the low-carbon economy, the topics of effective resource-acquiring methods have become the focus of green transformation for advanced manufacturing and modern service industries. Leasing activity plays an important role in establishing a low-carbon economy because it can help enterprises to reduce resource waste and promote reasonable capital flow. However, the uncertainty of leasing activity gives it an obvious online decision-making characteristic. Furthermore, the uncertainty is aggravated by drastic changes in the external economic environment, especially for those enterprises renting scarce resources. This paper studies an online leasing problem for a scarce resource that has double uncertainties in both price and usage time. An optimal online leasing strategy *ON* was then proposed. Experimental results on randomly generated instances verify the favorable efficiency of the proposed strategy under average-case scenarios. Furthermore, sensitivity analysis results indicate that the stable supply state and a low price-to-rent ratio are helpful for leasing activities to further expand positive influences on green transformation for manufacturing and service industries.

Based on the research results, some management enlightenments are put forward. First, the extreme price increase range caused by the unstable supply deteriorates the operational situation for enterprises seeking resources from the leasing market. Governments should maintain market supply and stabilize the overall price level to guide the healthy development of the leasing market. In addition, leasing companies should also establish reliable relationships with adequate suppliers to assure a stable supply. Second, a high price-to-rent ratio also deteriorates the operational situation under uncertainties. Hence, macroeconomic policies to control the price-to-rent ratio are another significant contribution to the development of the leasing market. In addition, for enterprises acquiring scarce resources with a high price-to-rent ratio, it is particularly important to eliminate the negative influence of uncertainties on resource acquisition decision-making. Finally, the challenges of the scarce resource supply have a significant impact on the leasing decision-making process. The proposed online strategy *ON* is proven to be the optimal online strategy and achieves good performances under average-case scenarios.

However, more complex environments exist in the actual operation of scarce resource leasing. For future research, it would be interesting to introduce interest rates and more general leasing types (e.g., financial leases) into the model to illustrate scenarios that are further closer to reality. Moreover, the demand quantity of the scarce resource studied in our model is assumed to be fixed each time. Hence, the uncertain demand quantity can also be considered in future research.

## Figures and Tables

**Table 1:** Parameters used in the online leasing problem for scarce resources.

Parameters	Description
*P*	price-to-rent ratio
*α*	price increase range from the regular supply state to the tight supply state
*C_ON_*	costs for using the resource produced by an online strategy *ON*
*C_opt_*	costs for using the resource produced by the optimal offline strategy
*ρ*	competitive ratio of the online strategy *ON*
*r_t_*	rental fee of the scarce resource at time *t*
*R_t_*	cumulative amount spent on rental fees before time *t*
*L*	the length of the scarce resource usage time
*λ*	a threshold parameter used in the online strategy *ON*

## Data Availability

Not applicable.
